# Lipid Metabolism and Breast Cancer: A Narrative Review of the Prognostic Implications and Chemotherapy-Induced Dyslipidemia

**DOI:** 10.3390/life15050689

**Published:** 2025-04-23

**Authors:** Ionut Flaviu Faur, Amadeus Dobrescu, Ioana Adelina Clim, Paul Pasca, Cosmin Burta, Marco Marian, Dan Brebu, Andreea-Adriana Neamtu, Vlad Braicu, Talpai Tamas, Ciprian Duta, Bogdan Totolici

**Affiliations:** 1 IInd Surgery Clinic, Timisoara Emergency County Hospital, 300723 Timisoara, Romania; flaviu.faur@umft.ro (I.F.F.); dobrescu.amadeus@umft.ro (A.D.); paul.pasca@umft.ro (P.P.); marian.marco@umft.ro (M.M.); brebu.dan@umft.ro (D.B.); braicu.vlad@umft.ro (V.B.); duta.ciprian@umft.ro (C.D.); 2 X Department of General Surgery, “Victor Babes” University of Medicine and Pharmacy Timisoara, 300041 Timisoara, Romania; 3 Multidisciplinary Doctoral School, “Vasile Goldiș” Western University of Arad, 310025 Arad, Romania; 4 Doctoral School of Medicine, “Victor Babes” University of Medicine and Pharmacy Timisoara, Eftimie Murgu Square 2, 300041 Timisoara, Romania; 5 Faculty of Medicine, “Victor Babes” University of Medicine and Pharmacy Timisoara, Eftimie Murgu Square 2, 300041 Timisoara, Romania; mihai.burta@student.umft.ro (C.B.); talpai.tamas@umft.ro (T.T.); 6 Faculty of Pharmacy, “Victor Babes” University of Medicine and Pharmacy Timisoara, Eftimie Murgu Sq., Nr. 2, 300041 Timisoara, Romania; andreea.neamtu@umft.ro; 7 IIIrd Surgery Clinic, Timisoara Emergency County Hospital, 300723 Timisoara, Romania; 8 Ist Clinic of General Surgery, Arad County Emergency Clinical Hospital, 310158 Arad, Romania; totolici.bogdan@uvvg.ro; 9 Department of General Surgery, Faculty of Medicine, “Vasile Goldiș” Western University of Arad, 310025 Arad, Romania

**Keywords:** breast cancer, neoadjuvant chemotherapy, chemotherapy-induced dyslipidemia, LDL-C, HDL-C

## Abstract

**Introduction:** Lipid metabolism plays a crucial role in breast cancer’s progression, treatment response, and prognosis. Alterations in triglycerides (TGs), low-density lipoprotein cholesterol (LDL-C), and high-density lipoprotein cholesterol (HDL-C) have been implicated in tumor aggressiveness and chemotherapy outcomes. This review examines the relationship between dyslipidemia and breast cancer, with a focus on chemotherapy-induced lipid alterations and their prognostic significance. **Methods:** A comprehensive literature search was conducted in PUBMED, Web of Science, and Google Scholar, identifying 108 unique studies. After applying the inclusion criteria, 21 studies were selected for analysis, covering lipid profile changes before, during, and after chemotherapy, as well as their impact on treatment response and clinical outcomes. **Results:** Breast cancer patients exhibited lower baseline TC, TG, and LDL-C levels compared to healthy controls; however, chemotherapy significantly increased these markers while decreasing HDL-C from 1.1 to 0.9 mmol/L. The incidence of dyslipidemia rose from 42.98% pre-treatment to 58.28% post-treatment. Chemotherapy-induced lipid alterations were most pronounced in anthracycline- and taxane-based regimens, leading to a 38% increase in TGs and a 23% reduction in HDL-C. While some studies reported that lipid levels normalized post-treatment, others indicated persistent dyslipidemia up to 12 months later. High baseline HDL-C was associated with a better chemotherapy response, whereas elevated TGs and LDL-C correlated with increased tumor aggressiveness, lower pathological complete response rates, and a higher relapse risk. Patients with persistently high post-treatment TGs had significantly worse disease-free survival, with a 30% relapse rate compared to 18% in those with normal TG. Preliminary evidence suggests that lipid-lowering therapies, such as statins, may offer therapeutic benefits in breast cancer by targeting the cholesterol synthesis pathways involved in tumor growth, though further clinical trials are required. **Conclusions:** Dyslipidemia is a key metabolic factor influencing breast cancer’s progression, treatment response, and long-term prognosis. Chemotherapy-induced lipid alterations may persist, increasing cardiovascular risk and potentially affecting therapeutic efficacy. Routine lipid monitoring and metabolic interventions could enhance treatment outcomes and survivorship. Future research should focus on developing lipid-targeted strategies to optimize breast cancer management.

## 1. Introduction

Breast cancer is the most common malignancy in women and a leading cause of cancer-related mortality worldwide [1,2,3]. Despite advances in early detection and targeted therapies, challenges remain in predicting treatment responses, managing side effects, and addressing outcome variations across molecular subtypes [4,5,6]. Understanding the biological mechanisms driving the progression of tumors and treatment response is key to improving outcomes [1].

Lipid metabolism has emerged as a critical area in breast cancer research. Cancer cells rely on altered lipid metabolism to meet their heightened energy demands, support rapid proliferation, and promote survival [7,8,9]. Dysregulated lipid profiles, such as elevated triglycerides and low-density lipoprotein cholesterol (LDL-C) with reduced high-density lipoprotein cholesterol (HDL-C), are frequently observed in breast cancer patients [1,10,11,12]. These changes are not incidental but driven by the metabolic needs of cancer cells, which use lipids for membrane synthesis, energy production, and signaling pathways that enhance tumor growth and metastasis [13,14].

The tumor microenvironment further emphasizes the role of lipids, as adipocytes in breast tissue provide the fatty acids and adipokines that fuel the progression of tumors. Lipid droplets within cancer cells have been linked to increased invasiveness and therapy resistance, highlighting lipid metabolism as both a marker and driver of disease [1,14,15,16].

Altered lipid profiles hold promise as non-invasive biomarkers for diagnosis, prognosis, and treatment monitoring. Additionally, targeting lipid metabolism offers potential therapeutic strategies, such as disrupting fatty acid synthesis or oxidation, to selectively impair cancer cells [15,17,18]. Understanding these metabolic changes can also help address the long-term effects of treatments like chemotherapy, which may exacerbate lipid dysregulation and increase comorbidities.

This review explores the relationship between lipid metabolism and breast cancer, with a particular focus on chemotherapy-induced dyslipidemia and its prognostic significance. By analyzing current evidence, we aim to clarify how lipid alterations impact cancer’s progression and treatment outcomes, highlighting potential implications for personalized therapy and metabolic interventions.

## 2. Materials and Methods

A systematic search was conducted in PubMed, Web of Science, and Google Scholar for studies published between 2000 and 2025. The search terms used included ‘breast cancer’, ‘dyslipidemia’, ‘chemotherapy-induced lipid alterations’, and ‘lipid metabolism’. Articles were included if they evaluated the impact of dyslipidemia on breast cancer’s prognosis or treatment outcomes. Studies without clinical data or focusing only on experimental models were excluded.

In addition to the primary database search, reference lists of all included studies and existing systematic reviews were manually screened to identify additional trials not indexed in the databases. Conference proceedings and grey literature databases were also reviewed to minimize publication bias and include findings from sources outside traditional peer-reviewed journals.

Following the initial search, 108 unique articles were identified. A preliminary review of the titles and abstracts excluded 89 studies that did not meet the inclusion criteria, leaving 19 articles for full-text review. During data abstraction, two additional relevant references were identified, increasing the total to 21 articles for comprehensive evaluation.

**Figure 1:**
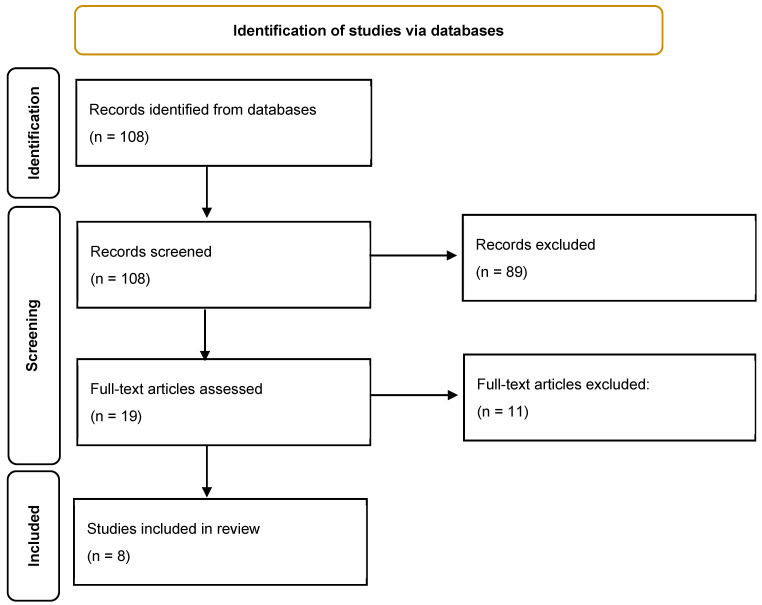
Flow diagram of study selection.

To be eligible for inclusion, the studies were required to meet the following criteria: be published in English, focus on women diagnosed with breast cancer, investigate the correlation between dyslipidemia and neoadjuvant chemotherapy (NAC) outcomes in breast cancer patients, and have reported at least one of the following: total cholesterol (TC), LDL-C, HDC-,c triglycerides (TGs), apolipoporteins A-1, A-2, B, C-2, C-4 (apoA-1, apoA-2, apoB, apoC-2, apoC-4). Ultimately, 8 articles met these criteria and were included in the final analysis (detailed in Figure 1 and Table 1).

## 3. Results

### 3.1. Chemotherapy-Induced Dyslipidemia: Evidence from Clinical Cohorts

Li et al. (2018) conducted a retrospective cohort study on 1054 breast cancer patients and observed a significant increase in TC, TGs, LDL-C following chemotherapy [19]. The prevalence of dyslipidemia rose from 42.98% before chemotherapy to 58.28% after treatment, with LDL-C levels increasing from 2.2 mmol/L pre-treatment to 2.8 mmol/L post-treatment (*p* < 0.001) and TG levels rising by 38%. In contrast, HDL-C levels declined significantly, from 1.1 mmol/L to 0.9 mmol/L, indicating chemotherapy-induced metabolic stress and disrupted lipid homeostasis.

Tian et al. (2019) provided further insight through a prospective cohort study involving 805 women with early-stage breast cancer, tracking lipid changes at multiple time points: before chemotherapy, at the final cycle of treatment, and six months post-treatment [20]. Similar to Li et al. [19], this study documented significant increases in TGs (from 1.4 mmol/L to 1.8 mmol/L, *p* < 0.001) and LDL-C (from 2.2 mmol/L to 2.7 mmol/L, *p* < 0.001) during chemotherapy. However, Tian et al. reported that these lipid changes were largely transient, with lipid levels returning to baseline within six months post-treatment. Interestingly, age appeared to influence chemotherapy-induced lipid alterations, with younger patients (aged 20–40 years) experiencing a 45% increase in TGs and a 30% increase in LDL-C levels, compared to a milder 25% and 15% increase in patients aged 41–65 years, respectively. These findings suggest that younger patients may be more metabolically susceptible to chemotherapy-induced lipid fluctuations, possibly due to differences in hormonal status, lipid metabolism, or treatment response.

Xu et al. (2020) conducted a longitudinal study on 159 newly diagnosed breast cancer patients, finding that lipid alterations persisted for at least 12 months post-chemotherapy, challenging the notion that dyslipidemia is merely a transient side effect [23]. Their study reported that TG levels remained elevated at 1.5 mmol/L one year after treatment, while LDL-C levels increased from 2.0 mmol/L at baseline to 2.3 mmol/L at 12 months post-chemotherapy (*p* < 0.05). The prevalence of dyslipidemia increased from 41.5% pre-treatment to 54.1% at one year, suggesting that chemotherapy-induced metabolic disruptions may contribute to long-term cardiovascular risk in breast cancer survivors. Additionally, postmenopausal patients (>50 years old) exhibited the most pronounced lipid alterations, likely due to the absence of estrogen’s protective effects on lipid metabolism.

He et al. (2020) conducted a retrospective study on 1934 breast cancer patients, evaluating the impact of different chemotherapy regimens on lipid profiles [22]. Their study found that TG, TC, and LDL-C levels increased significantly across all chemotherapy regimens, while HDL-C showed a notable decrease post-treatment. Specifically, TG levels rose from 1.5 mmol/L to 1.9 mmol/L (*p* < 0.001), while HDL-C decreased from 1.3 mmol/L to 1.0 mmol/L (*p* < 0.001). Among the regimens analyzed, anthracycline-plus-taxane chemotherapy caused the most severe lipid alterations, with TGs increasing by 38% and HDL-C decreasing by 23%, while fluorouracil-based regimens had a milder impact (TG and HDL-C changes limited to 12% and 8%, respectively, *p* < 0.05). The study also noted more severe lipid alterations in premenopausal patients, with TG levels increasing by 48% compared to a 25% increase in postmenopausal patients, reinforcing the need for lipid monitoring, especially in high-risk patient groups receiving anthracycline- and taxane-based regimens.

While chemotherapy-induced dyslipidemia has been widely documented, its clinical implications remain a subject of investigation. In particular, recent studies suggest that lipid alterations may not only reflect metabolic disruptions but also influence chemotherapy response. Understanding these associations could provide valuable insights into potential predictive biomarkers for treatment efficacy.

### 3.2. Predictive Role of Lipid Alterations in Chemotherapy Response

Qu et al. (2020) evaluated the predictive value of lipid profiles in the NAC response of 533 breast cancer patients [21]. Their study found that higher baseline HDL-C levels (≥1.305 mmol/L) were associated with better pathological complete response (pCR) rates (*p* = 0.007), while low TG levels (<1.155 mmol/L) predicted improved NAC outcomes. A ROC analysis demonstrated strong predictive values for TGs and HDL-C in the NAC response, with an AUC of 0.78 for TGs and 0.81 for HDL-C, supporting the clinical utility of lipid markers in guiding chemotherapy strategies.

Goto et al. (2023) explored the relationship between lipid metabolism, immune activity, and the chemotherapy response in 327 breast cancer patients, focusing on triple-negative breast cancer (TNBC) [24]. They found that TG levels increased significantly post-NAC in TNBC patients, rising from 1.2 mmol/L to 1.8 mmol/L (*p* = 0.049). Surprisingly, patients with higher post-treatment TG levels had improved overall survival (OS), with a 5-year OS rate of 78% compared to 65% in those with stable or decreasing TG levels (*p* = 0.014).

Beyond its role in the chemotherapy response, dyslipidemia may have lasting consequences on disease progression and patient survival. Investigating the long-term metabolic impact of lipid alterations could help determine whether dyslipidemia serves as a prognostic factor for breast cancer outcomes.

### 3.3. Long-Term Implications of Dyslipidemia in Breast Cancer’s Prognosis

Ma et al. (2023) found that pre-treatment dyslipidemia was associated with lower axillary pCR rates and poorer disease-free survival (DFS) in 312 breast cancer patients receiving NAC [25]. Post-treatment, patients with persistently high TG levels exhibited a 30% relapse rate, compared to 18% in those with normal TG levels (*p* < 0.05). A Cox regression analysis identified high TG levels as an independent predictor of relapse (HR = 1.896, *p* = 0.029), reinforcing the necessity of managing lipid abnormalities throughout the treatment course.

Santana et al. (2024) conducted an in-depth proteomic analysis of HDL-C in 141 newly diagnosed breast cancer patients, comparing their profiles to those of 143 matched healthy controls [4]. Their study identified significant molecular differences in HDL-C composition across different breast cancer subtypes, particularly in TNBC. The patients exhibited significantly lower levels of key apolipoproteins, including apoA-1, apoA-2, apoC-2, and apoC-4, compared to luminal A, luminal B, and HER2-positive breast cancer patients (*p*-values ranging from 0.004 to 0.057).

ApoA-1, a crucial protein in reverse cholesterol transport and anti-inflammatory pathways, was reduced by approximately 45% in TNBC cases compared to healthy controls, while apoA-2 levels were also significantly lower. Conversely, inflammatory proteins, such as complement C3, complement C9, complement factor B, and complement 4B, were significantly elevated in TNBC patients, suggesting a shift toward a pro-inflammatory HDL-C phenotype in aggressive breast cancer.

These proteomic changes correlated with the prognosis and patient survival, as patients in the lowest quartile of the apoA-1 expression had a 5-year overall survival rate of only 58%, compared to 82% in those with higher apoA-1 levels (*p* < 0.01). Similarly, patients with elevated complement system proteins (C3, C9) had a higher risk of recurrence and metastatic spread.

These findings suggest that HDL function in breast cancer is influenced not only by its cholesterol content but also by the specific composition of its associated proteins. This altered HDL-C phenotype may promote immune evasion, increase oxidative stress, and enhance its metastatic potential, particularly in aggressive subtypes such as TNBC.

### 3.4. Key Findings

The comprehensive analysis of current literature reveals several critical insights into the interplay between lipid metabolism and breast cancer.

### 3.5. Chemotherapy-Induced Dyslipidemia

Multiple studies have documented significant alterations in lipid profiles among breast cancer patients undergoing chemotherapy [4,20,21,23,24,25,26]. Notably, anthracycline-based regimens have been associated with the decreased expression of the ATP-binding cassette transporter A1 (ABCA1) and apolipoprotein A1 (apoA1), leading to reduced high-density lipoprotein cholesterol (HDL-C) levels. Taxane-based therapies, such as paclitaxel, have been linked to reductions in HDL-C and increases in lipid peroxidation products [4]. These lipid disturbances may contribute to the heightened cardiovascular risk observed in breast cancer survivors.

### 3.6. Transient vs. Persistent Dyslipidemia Post-Chemotherapy

The temporal nature of chemotherapy-induced dyslipidemia remains a subject of debate. Some studies suggest that lipid abnormalities are transient, normalizing after the completion of chemotherapy. Conversely, other research indicates that dyslipidemia may persist long after treatment cessation, potentially exacerbating long-term cardiovascular risk. This discrepancy underscores the necessity for ongoing lipid monitoring in breast cancer patients, both during and after chemotherapy [20,21,22,23,24,25,26].

### 3.7. Lipid Profiles as Prognostic Indicators

TG and LDL-C levels have been correlated with increased tumor aggressiveness and poorer clinical outcomes. These findings suggest that lipid profiles could serve as valuable prognostic biomarkers, aiding in the stratification of breast cancer patients and the tailoring of therapeutic interventions [20,21,22,23,24,25,26].

### 3.8. Impact of Lipid-Lowering Therapies

The potential therapeutic benefits of lipid-lowering agents, such as statins, in breast cancer management have garnered research interest. Preliminary findings indicate that statins may exert anti-tumor effects beyond their lipid-lowering properties, possibly through the inhibition of cholesterol synthesis pathways critical for tumor cell proliferation. However, further research is warranted to elucidate the efficacy and safety of integrating lipid-lowering therapies into breast cancer treatment protocols [20,21,26,27,28,29,30].

## 4. Discussion

### 4.1. Integration of Findings and Mechanistic Interpretations

The reviewed studies collectively underscore the significant role of lipid metabolism in breast cancer’s progression and treatment response. Notably, several investigations have reported elevated TG and LDL-C levels in patients undergoing chemotherapy, suggesting a potential link between dyslipidemia and adverse clinical outcomes [13,22,23,25,31]. For instance, Li et al. observed a significant increase in TG and LDL-C levels post-chemotherapy, implicating these alterations in heightened tumor aggressiveness [26]. Conversely, some studies have documented lower baseline lipid levels in breast cancer patients compared to healthy controls, raising questions about the temporal relationship between dyslipidemia and cancer development.

These discrepancies may be attributed to variations in study design, patient populations, and methodological approaches. Potential confounding factors, such as obesity, dietary habits, and genetic predispositions, warrant careful consideration. Obesity, in particular, is associated with both dyslipidemia and increased breast cancer risk, potentially confounding the observed associations. Furthermore, dietary patterns rich in saturated fats have been linked to altered lipid profiles and may influence cancer’s progression. Genetic factors, including polymorphisms in lipid metabolism-related genes, could also modulate individual susceptibility to dyslipidemia and its impact on cancer outcomes [14,32,33,34,35].

Mechanistically, dyslipidemia may promote the progression of tumors through several pathways. Elevated LDL-C can enhance cell membrane synthesis, providing structural support for rapidly proliferating cancer cells. Additionally, increased TG levels may serve as an energy reservoir, fueling tumor growth. Altered lipid profiles can also modulate the signaling pathways involved in cell proliferation and apoptosis, further contributing to cancer’s progression. Understanding these mechanisms is crucial for developing targeted interventions aimed at normalizing lipid metabolism to improve clinical outcomes in breast cancer patients [10,34,35,36,37,38].

### 4.2. Prognostic Role of Dyslipidemia in Breast Cancer

Dyslipidemia has increasingly been recognized as an essential factor influencing breast cancer’s prognosis, particularly among patients undergoing NAC. Multiple studies have indicated that elevated levels of LDL-C and triglycerides (TGs) are associated with worse clinical outcomes, including a higher tumor burden, increased rates of recurrence, and reduced disease-free survival, while HDL-C has shown potential protective effects [4,20,21,24,26,39,40,41,42]. A study by Rodrigues dos Santos et al. demonstrated that breast cancer patients with elevated LDL-C at diagnosis had significantly larger tumors, higher histological grades, increased Ki-67 proliferation indices, and a more frequent HER2-positive status, suggesting that systemic cholesterol plays a role in cancer’s progression. The authors further observed that high LDL-C levels at diagnosis were linked to reduced DFS, reinforcing its prognostic significance. These findings align with earlier research highlighting the impact of lipid metabolism on the progression of tumors and the potential for targeting cholesterol pathways in breast cancer therapy [43,44].

Moreover, chemotherapy-induced dyslipidemia is a growing concern, as several studies have reported significant alterations in lipid profiles during and after treatment. Bicakli et al. found that adjuvant chemotherapy contributes to increased TG and LDL-C levels while reducing HDL-C, a pattern that persisted post-treatment [26]. This metabolic disruption has been linked to chemotherapy resistance and tumor adaptation. These findings suggest that lipid metabolism alterations could contribute to NAC resistance, making lipid-targeted therapies a promising avenue for overcoming chemoresistance.

The relationship between HDL-C and breast cancer’s prognosis remains complex. While higher HDL-C levels are generally associated with improved outcomes, studies have also indicated potential pro-tumorigenic effects under specific conditions. For instance, Kim et al. reported that decreased HDL-C was significantly associated with oxidative stress and increased levels of pro-inflammatory cytokines, such as the tumor necrosis factor-alpha (TNF-α) and interleukin-6 (IL-6), both of which play crucial roles in cancer’s progression [45]. This inflammatory response could contribute to a poorer prognosis, particularly in postmenopausal patients, where dysregulated lipid metabolism is more prevalent.

TGs have also emerged as a crucial prognostic marker in breast cancer. Several studies have shown that elevated TG levels at baseline or following NAC correlate with an increased recurrence risk. In a study analyzing lipid profile alterations during chemotherapy, it was observed that patients with persistently high TG levels post-treatment had a significantly higher risk of developing distant metastases compared to those with normalized lipid levels [46]. These findings underscore the importance of routine lipid monitoring and early intervention strategies to mitigate adverse metabolic effects during and after chemotherapy.

The impact of dyslipidemia extends beyond its role in the progression of tumors to its influence on treatment response. Research has indicated that lipid abnormalities may impair chemotherapy efficacy through multiple mechanisms, including alterations in drug pharmacokinetics, the modulation of apoptotic pathways, and enhancement of pro-survival signaling in cancer cells. Tőkés et al. noted that breast cancer subtypes exhibit distinct lipid metabolism profiles, with hormone receptor-positive (HR+) tumors relying on a balance between fatty acid synthesis and oxidation, while TNBCs show an increased dependence on exogenous fatty acids [47]. These metabolic distinctions suggest that lipid-targeted interventions may need to be tailored to specific breast cancer subtypes.

In addition to its implications for breast cancer’s progression and treatment resistance, dyslipidemia has been linked to cardiovascular complications in breast cancer patients undergoing NAC [48,49,50,51]. Several studies have demonstrated that anthracycline- and taxane-based chemotherapy regimens frequently induce dyslipidemic changes, contributing to an increased risk of cardiovascular disease in long-term breast cancer survivors. Research has shown that dyslipidemia worsens following these chemotherapy treatments, emphasizing the need for continuous lipid monitoring. Additionally, evidence suggests that anthracyclines may have atherogenic effects, further linking chemotherapy-induced lipid alterations to a heightened cardiovascular risk [25,52]. This highlights the need for an integrated approach to cancer care that includes a cardiovascular risk assessment and lipid management.

Given the growing body of evidence linking dyslipidemia to breast cancer’s prognosis, therapeutic strategies targeting lipid metabolism are being explored. Statins, widely used for cholesterol-lowering purposes, have demonstrated potential anti-cancer effects through the inhibition of cholesterol biosynthesis, induction of apoptosis, and suppression of inflammatory pathways [53]. Some retrospective analyses have suggested that statin use is associated with improved breast cancer outcomes, particularly in hormone receptor-positive subtypes [27,28,54]. However, further randomized controlled trials are needed to establish their role in standard oncology practice.

### 4.3. Limitations and Future Directions

While this review provides valuable insights into the interplay between lipid metabolism and breast cancer, several limitations must be acknowledged.

First, the majority of the included studies are observational in nature, precluding causal inferences. Selection bias is a concern, as many studies utilized hospital-based cohorts, which may not be representative of the broader patient population. Additionally, potential confounding factors, such as the body mass index (BMI), dietary intake, and physical activity levels, were not consistently controlled across the studies, potentially influencing lipid profiles and cancer outcomes [55].

Publication bias may also be present, as studies with significant findings are more likely to be published, skewing the available evidence. Furthermore, the heterogeneity in the study designs, lipid measurement techniques, and definitions of dyslipidemia complicates the synthesis of findings.

Future research should aim to address these limitations by employing prospective cohort designs with larger, more diverse populations. Standardized methodologies for lipid assessment and uniform definitions of dyslipidemia are essential to facilitate comparability across studies. Moreover, mechanistic studies exploring the causal pathways linking lipid metabolism to breast cancer’s progression are warranted. Investigations into the impact of the interventions targeting lipid metabolism, such as lifestyle modifications and pharmacological agents, on breast cancer outcomes could provide valuable insights for clinical practice.

## 5. Conclusions

Dyslipidemia significantly influences breast cancer’s progression, prognosis, and treatment response, with elevated TG and LDL-C levels associated with worse clinical outcomes and chemotherapy resistance. Chemotherapy-induced lipid alterations, whether transient or persistent, may contribute to metabolic imbalances and an increased cardiovascular risk in survivors. Integrating lipid monitoring into oncologic care and exploring lipid-targeted therapies, such as statins, could enhance treatment efficacy and long-term patient outcomes. Future research should focus on refining lipid-based prognostic models and developing targeted metabolic interventions to improve breast cancer management.

## Figures and Tables

**Table 1:** Summary of studies on Lipid Metabolism in Breast Cancer Patients.

Article (Year)	Study Design	Sample Size	Breast Cancer Subtype	Lipid Parameters Assessed	Main Findings	Chemotherapy Regimen	Follow-Up Duration	Key Outcome
Li et al. (2018) [19]	Retrospective cohort	1054 BC patients, 2483 controls	Mixed	TC, TGs, LDL-C, HDL-C	BC patient had significantly lower TC, TGs, LDL-C, and HDC-c than controls (*p* < 0.05). Dyslipidemia worsened post-chemotherapy, with TGs and LDL-C increasing while HDL-C decreased.	Various	Not specified	Dyslipidemia incidence increased from 42.98% to 58.28% after chemotherapy.
Tian et al. (2019) [20]	Prospective cohort	805	Mixed	TC, TGs, LDL-C, HDL-C	Chemotherapy-induced lipid alterations were transient, with TC and LDL-C increasing during treatment but returning to baseline after 6 months. Younger patients showed greater lipid fluctuations.	Various	6 months	Chemotherapy-induced dyslipidemia is temporary in most patients.
Qu et al. (2020) [21]	Retrospective	533	ER+	TGs, HDL-C	High pre-NAC HDL-C (≥1.305 mmol/L) was an independent predictor of better chemotherapy response (*p* = 0.007). Low TGs (<1.155 mmol/L) also predicted improved NAC response.	NAC	Not specified	Lipid profiles may serve as biomarkers for NAC response.
He et al. (2020) [22]	Retrospective	1934	Mixed	TC, TGs, LDL-C, HDL-C	TGs increased by 38%, HDL-C decreased by 23% after chemotherapy. FEC had milder lipid changes than taxane-based regimens. Postmenopausal women had less severe lipid alterations.	AC-T, EC-T, TC, FEC	Not specified	Chemotherapy significantly alters lipid profiles, with taxane-based regimens showing more pronounced effects.
Xu et al. (2020) [23]	Longitudinal	159	Mixed	TC, TGs, LDL-C, HDL-C	TGs increased from 1.3 to 1.6 mmol/L post-chemotherapy and remained elevated at 12 months (*p* < 0.05). LDL-C also remained high, while HDL-C was stable.	Various	12 months	Persistent dyslipidemia post-chemotherapy, requiring long-term lipid management.
Goto et al. (2023) [24]	Prospective	327	TNBC	TGs, HDL-C, TC	Post-NAC TG increase correlated with improved survival (5-year OS: 78% vs. 65%, *p* = 0.014). High TC post-NAC was linked to better survival in TNBC patients (*p* = 0.014).	NAC	5 years	Lipid metabolism influences immune activity and survival in TNBC.
Ma et al. (2023) [25]	Retrospective	312	Mixed	TC, TGs, LDL-C	Pre-treatment dyslipidemia was associated with lower pCR rates and higher relapse (30% vs. 18%, *p* < 0.05). Full-course lipid levels correlated with DFS (HR = 1.896, *p* = 0.029).	NAC	Not specified	High TG levels independently predicted poor DFS.
Santana et al. (2024) [4]	Cross-sectional	141 BC patients, 103 controls	Mixed	HDL-C, ApoA-1, ApoA-2, ApoC-2, Apo C-4	TNBC patients had lower ApoA-1 and ApoA-2 levels than other subtypes, suggesting altered HDL proteomics. Higher HDL-associated proteins correlated with tumor stage.	Not applicable	Not applicable	HDL composition differs by BC subtype and may serve as a biomarker.
